# Crayfish hemocytes develop along the granular cell lineage

**DOI:** 10.1038/s41598-021-92473-9

**Published:** 2021-06-23

**Authors:** Fang Li, Zaichao Zheng, Hongyu Li, Rongrong Fu, Limei Xu, Feng Yang

**Affiliations:** 1 Key Laboratory of Marine Genetic Resources, Third Institute of Oceanography, Ministry of Natural Resources, 184# Daxue Road, Xiamen, 361005 China; 2 Fujian Key Laboratory of Marine Genetic Resources, Xiamen, 361005 China

**Keywords:** Haematopoiesis, Differentiation

## Abstract

Despite the central role of hemocytes in crustacean immunity, the process of hemocyte differentiation and maturation remains unclear. In some decapods, it has been proposed that the two main types of hemocytes, granular cells (GCs) and semigranular cells (SGCs), differentiate along separate lineages. However, our current findings challenge this model. By tracking newly produced hemocytes and transplanted cells, we demonstrate that almost all the circulating hemocytes of crayfish belong to the GC lineage. SGCs and GCs may represent hemocytes of different developmental stages rather than two types of fully differentiated cells. Hemocyte precursors produced by progenitor cells differentiate in the hematopoietic tissue (HPT) for 3 ~ 4 days. Immature hemocytes are released from HPT in the form of SGCs and take 1 ~ 3 months to mature in the circulation. GCs represent the terminal stage of development. They can survive for as long as 2 months. The changes in the expression pattern of marker genes during GC differentiation support our conclusions. Further analysis of hemocyte phagocytosis indicates the existence of functionally different subpopulations. These findings may reshape our understanding of crustacean hematopoiesis and may lead to reconsideration of the roles and relationship of circulating hemocytes.

## Introduction

In multicellular animals, blood cells (also called hemocytes, coelomocytes, or amebocytes) are the basic immune cells responsible for the detection and clearance of invading pathogens^1^. In some species, blood cells also participate in the transportation of oxygen and nutrition^2,3^. The production of new blood cells by hematopoiesis is critical to the functioning of the immune system and the body as a whole.

Innate immunity exists in both vertebrates and invertebrates. Recent evidence suggests that some immune cells and the proteins controlling their development are conserved among these animals^1,4,5^. Compared to the comprehensive understanding of hematopoiesis in higher vertebrates, what we know in invertebrates is limited. Most of our knowledge comes from research in insects^1,6,7^. In addition, there are some studies concerning blood cell formation in tunicates^8,9^, echinoderms^10,11^, mollusks^12,13^ and crustaceans^14–19^. Investigation of hematopoiesis in various species will help us understand how this critical process evolved in the animal kingdom.

Crustaceans are an extremely diverse group of arthropods that live mainly in aquatic environments^14,20^. Crustacean hemocytes are classified into three morphological types: granular cells/large granular cells (GCs), semigranular cells/small granular cells (SGCs), and hyaline cells (HCs)/agranular cells^21^, although the cell type composition varies among species. They are immune cells responsible for phagocytosis, encapsulation, nodule formation, melanization, and wound healing. In crayfish, GCs and SGCs are the predominant types of hemocytes, whereas HCs are very rare^14,22,23^. GCs are the main cells that store prophenoloxidase and clotting enzymes, while SGCs function in encapsulation^21^. Both GCs and SGCs are capable of phagocytosis, but they target different foreign particles^23^. HCs are thought to be immature hemocytes occasionally released from HPT under certain conditions^14^. The existence of subpopulations of the morphological types of hemocytes has been proposed in decapods based on their staining property^24–26^, gene expression pattern^27,28^, or immune function^23,29^, but with no in-depth study.

The hematopoietic tissue (HPT) of decapod crustaceans is a thin sheet of structure covering the dorsal side of the stomach^15,18,19,30^. The HPT is formed by lobules filled with hemocyte precursors. In crayfish, five morphological types of cells have been identified in the HPT. Type 1 and type 2 cells have been proposed to be less differentiated precursors for both GCs and SGCs; type 3 and 4 cells are precursors for GCs; and type 5 cells are precursors for SGCs^16^. Regulators involved in crayfish hematopoiesis have been identified. Astakine 1 (AST1), an important regulator of homeostasis, may function by directly stimulating the proliferation of HPT cells, promoting SGC differentiation^31^, and enhancing the release of new hemocytes^32,33^. At the same time, AST1 prevents HPT cells and hemocytes from undergoing apoptosis by upregulating the expression of crustacean hematopoietic factor (CHF)^28^. Moreover, some other molecules have been found to regulate crayfish hematopoiesis through AST1^34,35^. In addition, Astakine 2, a paralog of AST1, may play a role in GC maturation^17,36^. A hypothetical model has been proposed for hemocyte differentiation in crayfish^16,30^. In this model, a common progenitor gives rise to lineage-committed progenitors for SGCs and GCs, and these lineage-specific progenitors further develop into young SGCs and GCs. Similar two-lineage models for hemocyte differentiation have also been suggested in penaeid shrimp^18^ and lobster^37^. However, in these two reports, the authors also mentioned the possibility for sequential development from SGCs to GCs. There are preliminary studies indicating that the observed hemocyte types in crustaceans may be functional or developmental stages of one cell type^38,39^. These hypotheses are based mainly on morphological evidence; thus, the differentiation of crustacean hemocytes remains an open question.

Research in crayfish shows that there are no mature GCs in the lobules of HPT^30,40^, and prophenoloxidase, a marker gene for mature hemocytes, is not expressed in this tissue^22,40,41^. Thus, the final stage of GC differentiation is likely completed in the circulation. There are similar observations in penaeid shrimp and lobster^18,19^. Therefore, at least a portion of SGCs are immature GCs. Currently, we do not know how abundant these immature cells are and how they develop in the circulation. In addition, we recently found that over 60% of the hemocyte precursors in the HPT of crayfish can differentiate into GCs in vitro upon induction, and the immature GCs are very similar in morphology to circulating SGCs^40^. Therefore, it is likely that a large majority of hemocytes have a GC fate.

Here, to address these questions, we investigated the developmental process of hemocytes in crayfish by tracking newly produced cells and by allotransplantation of HPT cells and hemocytes. We propose that almost all crayfish hemocytes finally develop into GCs. The long-known SGCs and GCs may represent hemocytes at different developmental stages. These findings may reshape our understanding of crustacean hematopoiesis and extend our knowledge of hematopoiesis in invertebrates.

## Materials and methods

### Animals

Crayfish, *Cherax quadricarinatus*, were purchased from Zhangzhou Yuansentai Agriculture Technology Co., Ltd., (Zhangzhou, China) and Xiamen Jixiong Aquaculture Co., Ltd., (Xiamen, China). The crayfish were maintained in fresh water at 25 °C and fed daily with a commercial diet. The animals were acclimated for at least 10 days in the laboratory and determined to be free of white spot syndrome virus (WSSV) and decapod iridescent virus 1 (DIV1) before use. WSSV and DIV1 were detected by real-time fluorescent quantitative PCR using the White Spot Syndrome Virus (WSSV) Fluorescent Quantitative PCR Detection Kit and Decapod Iridescent Virus 1 (DIV1) Fluorescent Quantitative PCR Detection Kit (Xiamen Zhihuilianfeng Biotechnology Co., Ltd., Xiamen, China).

Only intermolt animals were used in the experiments. Male and female crayfish were used in the analysis of hemocyte mortality. Male crayfish were used in all the remaining experiments, since ovary development often occurred in the females during the long-term experiments, which could cause problems in handling and increased uncertainty in the experiments. Animals weighing 45 ± 5 g were used in the EdU incorporation experiments and the analysis of hemocyte mortality. In the transplantation experiments, animals weighing 70 ± 10 g were used as donors, and animals weighing 45 ± 5 g were used as recipients. We used large animals as donors since it was much easier to obtain enough cells for transplantation. In particular, animals weighing 32 ± 5 g were used as recipients for purified SGCs and GCs, since it was more difficult to obtain a large number of purified cells for transplantation.

All animal experiments in the study were approved by the Animal Care and Use Committee of Third Institute of Oceanography, Ministry of Natural Resources. All methods were performed in accordance with the relevant guidelines and regulations.

### Analysis of hemocyte mortality

Hemolymph (200 μL) was taken from each animal in the ventral hemocoel of the second abdominal segment with a sterile syringe preloaded with an equal volume of anticoagulant solution (26 mM sodium citrate, 30 mM citric acid, 100 mM glucose, 140 mM NaCl, pH 5.8). Propidium iodide (PI) was immediately added to the hemocyte suspension at a final concentration of 5 μg/mL. Cells were incubated at room temperature for 5 min. Half of the cells were analyzed by flow cytometry to measure the percentage of dead cells, and the other half were observed under a fluorescence microscope. To avoid the change in mortality during handling, each sample was analyzed within 15 min after collection, without any centrifugation. Twenty animals, 10 males and 10 females, were used in this experiment.

### Analysis of the rate of new hemocyte production

Crayfish were injected with 5-ethynyl-2′-deoxyuridine (EdU) (Thermo Fisher) at a dose of 15 μg/g body weight^42,43^. For the analysis of hemocytes, 200 μL of hemolymph was taken from each animal at different time points with a syringe preloaded with an equal volume of 8% paraformaldehyde. Samples were taken from 8 animals at each time point from day 1 to day 8. Samples were taken from > 4 animals at each time point within 24 h. For the analysis of HPT cells, HPT cells were isolated as previously described^31^ with some modification. In brief, the HPTs were incubated with 0.1% collagenase (type I and IV) (Sigma) in crayfish phosphate buffered saline (CPBS) (10 mM Na_2_HPO_4_, 10 mM KH_2_PO_4_, 150 mM NaCl, 10 μM CaCl_2_, 10 μM MnCl_2_, and 2.7 μM KCl, pH 6.8)^28,44^ at 30 °C for 30 min. The dissociated HPT cells were washed once with CPBS and collected by centrifugation at 300×*g* for 3 min at room temperature. Cells were resuspended in CPBS and passed through a cell strainer (70 μm), and an equal volume of 8% paraformaldehyde was added. Samples were taken from 8 animals at each time point from day 1 to day 8. Samples were taken from > 4 animals at each time point within 24 h.

After fixation at room temperature for 15 min and permeabilization with 0.5% Triton X-100 for 20 min, the cells were collected by centrifugation at 300×*g* for 3 min at room temperature and resuspended in reaction buffer in a Click-iT® EdU Alexa Fluor® 488 Flow Cytometry Assay Kit (Thermo Fisher). EdU was detected following the instructions, and the cells were then analyzed by flow cytometry and fluorescence microscopy.

### Tracking the development of newly produced hemocytes by EdU incorporation

Crayfish were injected with EdU at a dose of 15 μg/g body weight. Hemolymph was taken from the animals at 7, 14, 28, 42, and 56 days post-injection (dpi). Hemocytes were collected and EdU was detected as described above. Eight animals were used in this experiment.

### Preparation of cells for transplantation

#### Preparation of HPT cells

HPT cells were isolated as described above, resuspended in CPBS and passed through a cell strainer.

#### Preparation of total hemocytes

Hemolymph was taken from each animal using a sterile syringe preloaded with an equal volume of anticoagulant solution. Hemocytes were collected by centrifugation at 300×*g* for 3 min at room temperature and were ready for fluorescence labeling or purification of SGCs and GCs.

#### Purification of SGCs and GCs

SGCs and GCs were purified from circulating hemocytes as previously described^45^. In brief, 200 μL of hemocyte suspension (~ 1 × 10^7^ cells, in anticoagulant solution) was loaded onto a prechilled Percoll density gradient containing 0.2 mL 100% Percoll, 2 mL 65% Percoll, and 1 mL 20% Percoll (from bottom to top). Percoll was diluted with the anticoagulant solution. The cells were separated by centrifugation at 450×*g* for 30 min. SGCs formed a layer on the interface of 20% and 65% Percoll, while GCs formed a layer on the interface of 65% and 100% Percoll. Each cell layer was collected and diluted in 6–10 volumes of prechilled anticoagulant solution and harvested by centrifugation at 350×*g* for 5 min at 4 °C. The purity of SGCs and GCs was analyzed by flow cytometry^23^ and fluorescence microscopy.

### Cell label and transplantation

The cell concentration was measured using an Orfol's Moxi Z cell counter. Total hemocytes and purified SGCs/GCs were labeled using the Vybrant® CFDA SE Cell Tracer Kit (Thermo Fisher). In brief, 5 × 10^6^ cells were incubated with 1 mL of 10 μM CFDA SE in anticoagulant solution for 15 min at room temperature. After one wash with anticoagulant solution, the cells were resuspended in CPBS. HPT cells were labeled with CellTrace™ Yellow (Thermo Fisher). Cells were incubated with 10 μM CellTracer Yellow in CPBS at room temperature for 20 min (5 × 10^6^ cells in 1 mL solution) and collected by centrifugation at 300×*g* for 3 min at room temperature. The pellet was resuspended in CPBS and incubated for another 10 min.

For allotransplantation, the cell suspensions were injected into the ventral hemocoel of the second abdominal segment. We chose the ventral hemocoel as the injection site because the hemocoel is part of the circulating system and is easy to access. The material injected into the hemocoel can be rapidly transported throughout the body. For each animal, ~ 1 × 10^7^ total hemocytes, ~ 9 × 10^6^ HPT cells, ~ 6 × 10^6^ SGCs, or ~ 4 × 10^5^ GCs were transplanted. The yield of GCs was low in the purification assay, so fewer cells were transplanted. The total volume of cell suspension injected into each animal was 200 μL.

The experiments were performed twice. Each time, 5 animals were used in the transplantation experiment for HPT cells, total hemocytes, and SGCs. Since it was difficult to obtain purified GCs, GC transplantation was performed in 3 animals.

### Sample collection and analysis in the transplantation experiments

For animals transplanted with HPT cells and total hemocytes, hemolymph samples were taken each week, at 7, 14, 28, 42, 56, 70, 84, and 98 days post-transplantation (dpt). We did not present the data at 98 dpt for total hemocyte transplantation because nearly all the transplanted cells already became GCs at 84 dpt (see the results), and the percentage of allogenic cells was very low on day 98. Animals transplanted with SGCs and GCs were smaller than the animals used in other experiments. Thus, it was more difficult for them to have multiple samples taken. For this experiment, hemolymph samples were collected at 7, 28, 56, 70, and 84 dpt.

At each time point, hemolymph (150 μL for HPT cell and total hemocyte transplantation groups; 100 μL for SGC and GC transplantation groups) was taken from each animal as described above. After fixation at room temperature for 15 min, the cells were collected by centrifugation at 300×*g* for 3 min and resuspended in PBS (NaCl 136.89 mM; KCl 2.67 mM; Na2HPO4 8.1 mM; KH2PO4 1.76 mM, pH 7.3). The percentage of transplanted cells (fluorescence labeled) was analyzed by flow cytometry. The percentage of fluorescent GCs and SGCs was counted under a fluorescence microscope. At earlier time points, over 200 fluorescent cells were counted for each sample. At later time points, all the fluorescent cells in the whole sample were counted, but the total number was sometimes less than 200. Only cells filled with many large cytoplasmic granules were classified as GCs.

### Flow cytometry analysis

Flow cytometry analysis was performed with a BD FACSCalibur flow cytometer. Cells with fluorescence were identified based on FL1-H (for EdU-incorporated and CFDA SE-labeled cells) or FL2-H (for CellTrace™ Yellow-labeled cells and PI-stained cells) parameters.

For EdU incorporation experiments, hemocytes isolated from uninjected crayfish were stained as well and served as the negative control. For the transplantation assay, hemocytes taken from untransplanted crayfish were used as the negative control.

Ten thousand cells were analyzed for each sample.

### Primary culture of crayfish HPT cells and induction of GC differentiation

HPT cells were isolated as described above. The cells were resuspended in Leibovitz's L-15 medium, seeded in 48-well plates (4 × 10^5^/well), and grown at 27 °C. The next day, GC differentiation was induced using the induction medium (L-15 medium, 1% FBS, 20% *C. quadricarinatus* muscle extract)^40^. Half the culture medium was changed every 7 days.

### RNA isolation and RT-PCR analysis

Total RNA was isolated using an Eastep Super total RNA extraction kit (Promega). GCs and SGCs were purified as described above and lysed with RNA lysis buffer. Cultured HPT cells were lysed in-well. For each sample, ~ 8 × 10^5^ cells were lysed. Total RNA was extracted as instructed, and genomic DNA contamination was removed by DNase I treatment. First strand cDNA was synthesized using SuperScript III Reverse Transcriptase (Thermo Fisher Scientific) with the oligo dT_(18)_ primer. Marker gene expression was analyzed by PCR using the primers provided in Table S1. The reaction was performed as follows: initial denaturing at 94 °C for 3 min; 25–33 cycles of 94 °C for 30 s, 55 °C for 30 s, and 72 °C for 50 s; and a final extension at 72 °C for 5 min. The PCR products were analyzed by electrophoresis.

### In vivo﻿﻿ phagocytosis assay

An in vivo phagocytosis assay was performed as previously described^23^. Carboxylate-modified fluorescent microspheres, 1 μm yellow-green (505/515) (Thermo Fisher), were dialyzed against 0.9% NaCl and sonicated briefly before use. Fluorescent microspheres were resuspended in 0.9% NaCl and injected into the hemocoel of three crayfish at the base of the fifth walking leg (the ratio of particle to cell = 100:1). The total volume injected into each animal was 150 μL. Hemolymph (200 μL) was collected from each animal at 4 h post-injection (hpi), when the phagocytosis ratio (phagocytic hemocytes/total hemocytes) reached a plateau^23^. Hemocytes were fixed as described above, washed with PBS, and analyzed by fluorescence microscopy. Over 100 phagocytic cells were counted for each sample.

## Results

### The renewal rate of circulating hemocytes

**Figure 1:**
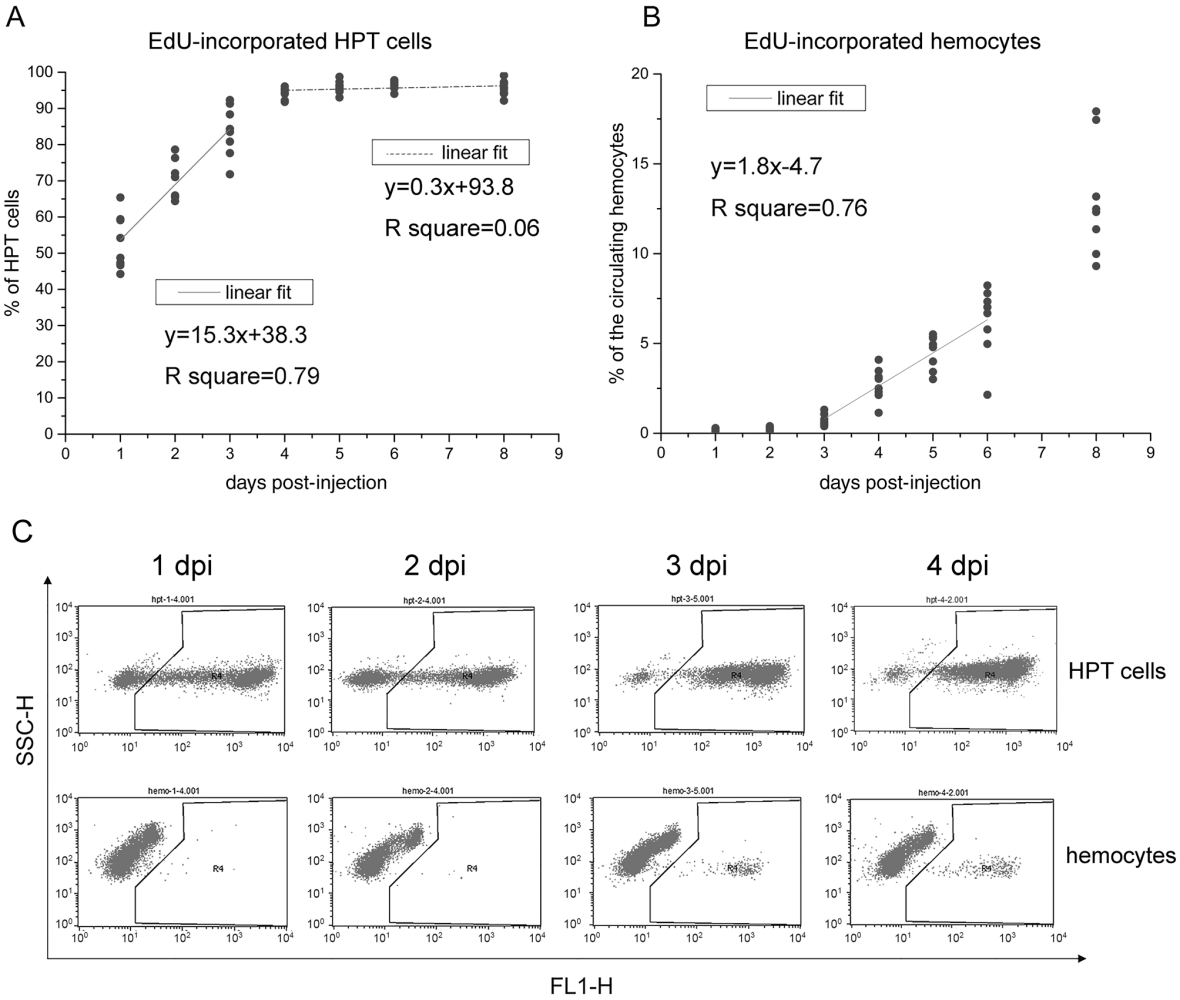
The renewal rate of circulating hemocytes. Crayfish were injected with one dose of EdU. HPT cells and circulating hemocytes were collected at different time points until 8 dpi. After fixation, cells were stained for EdU and analyzed by flow cytometry. Over 10,000 cells were analyzed for each sample. The percentages of EdU-incorporated cells in the HPT (**A**) and circulation (**B**) are shown with percentage-time scatter plots. Eight animals were analyzed at each time point. The relationships between time and EdU incorporation percentages were modeled using linear regression (1 ~ 3 dpi and 4 ~ 8 dpi for HPT cells; 3 ~ 6 dpi for circulating hemocytes). (**C**) Typical FL1-SSC scatterplots obtained in flow cytometry analysis (HPT cells, upper panel; hemocytes, lower panel). Cells in the right region of each plot are EdU-incorporated.

The renewal rate of circulating hemocytes is a critical parameter for hemocyte homeostasis, which is currently unclear in crustaceans. We measured the rate of new hemocyte production in crayfish by EdU incorporation. EdU was incorporated into the DNA of proliferating cells and transferred to their progeny. If the rate of new cell production was relatively stable, we would see a linear increase in EdU-incorporated cells in HPT. In the experiment, each animal was injected with one dose of EdU. HPT cells and circulating hemocytes were collected at different time points until 8 dpi and analyzed by flow cytometry and fluorescence microscopy. On average, there were ~ 20% EdU-incorporated cells in the HPT at 2 hpi (Fig. S1). The average percentage of EdU-incorporated cells remained < 30% until 10 hpi. From day 1 to day 3, the increase in EdU-incorporated cells in HPT was basically linear (y = 15.3x + 38.3, R^2^ = 0.79) (Fig. 1A). The linear phase began when all the proliferating cells were labeled and ended after all the cells in the tissue were labeled. Over 95% of the HPT cells became EdU-incorporated since day 4. However, there was always a small portion (< 5%) of cells in the tissue that did not incorporate EdU. Based on these data, the HPT produced ~ 15% new cells every day.

The circulating hemocytes of crayfish scarcely proliferate^41^. In the circulation, there were no (or extremely few < 0.3%) EdU-incorporated cells in the first two days. The EdU-incorporated hemocytes began to be released from HPT at 3 dpi, and increased in a linear manner from day 3 to 6 (y = 1.8x-4.7, R^2^ = 0.76) (Fig. 1B). We did not use the data of day 8 for fitting because the effects of repeated sampling increased over time. Figure 1C shows the typical flow cytometry results for both HPT cells and circulating hemocytes (FL1-SSC scatter plots). The fluorescent signal for EdU was detected in the FL1 channel. The spots in the right region of each plot were EdU-incorporated cells. These data indicated that ~ 1.8% of the circulating hemocytes were renewed every day.

In addition, we measured the mortality of circulating hemocytes by PI staining (Table S2). Based on the data from 20 animals varying from 28 to 60 g, including 10 females and 10 males, we found that under normal physical status, ~ 1% of the cells in the circulation were dead, and there was no obvious difference between males and females. However, these data represented only the portion of dead cells at that moment, and we could not directly measure daily mortality. Approximately 60% of these dead cells had a relatively intact structure (with the plasma membrane and nucleus), so we could distinguish GCs and SGCs. Among these cells, over 94% were GCs, indicating that GCs were the major senescent cells in circulation.

### New hemocytes belonging to the GC lineage take approximately a month to mature

**Figure 2:**
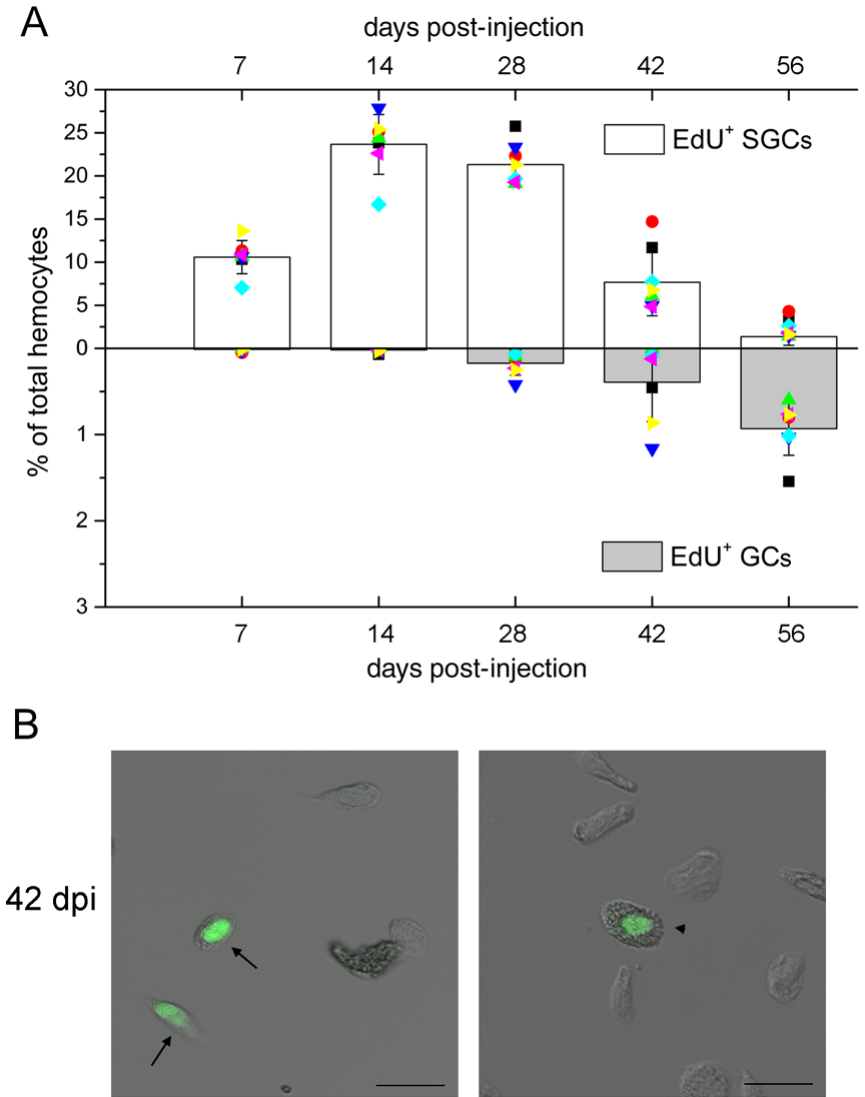
New hemocytes belonging to the GC lineage take approximately one month to mature. Crayfish were injected with one dose of EdU. Hemolymph was taken at different time intervals until 56 dpi for analysis. The percentages of EdU-incorporated (EdU^+^, green) GCs and SGCs in the total hemocytes were determined by flow cytometry and recorded until 56 dpi (**A**). Eight animals were used in each group. For each sample, over 10,000 cells were analyzed. Data from different individuals are indicated by different symbols. Images of EdU-incorporated hemocytes at 42 dpi are shown (**B**). EdU-incorporated SGCs are indicated with arrows, and EdU-incorporated GCs are indicated with arrowheads. Bar, 25 μm.

To track the development of newly produced hemocytes, crayfish were injected with EdU. The percentages of EdU-incorporated GCs and SGCs in the circulation were analyzed by flow cytometry^23^ and recorded until 56 dpi. HCs were not considered in this study because HCs could barely be found in the circulation by microscopy, and no hemocyte population corresponding to HCs could be identified by flow cytometry. As shown in Fig. 2, the EdU-labeled hemocytes represented ~ 10% of the circulating hemocytes at 7 dpi, slowly increased to 20~23% at 14~28 dpi, and then declined. In the first 2 weeks, the EdU-incorporated hemocytes were exclusively SGCs. Then, a very small number of EdU-labeled GCs (< 0.2% of the total hemocytes, < 1.0% of EdU-incorporated cells) appeared at 28 dpi. The percentage of EdU-incorporated cells dramatically declined after a month, and the highest number of EdU-incorporated GCs represented only ~ 0.9% of the total circulating hemocytes (43.9% of the EdU-incorporated hemocytes) at 56 dpi. These results suggested that new hemocytes were released from HPT in the form of SGCs, and those belonging to the GC lineage took approximately a month to mature.

### Approximately 60% of transplanted HPT cells develop into GCs

**Figure 3:**
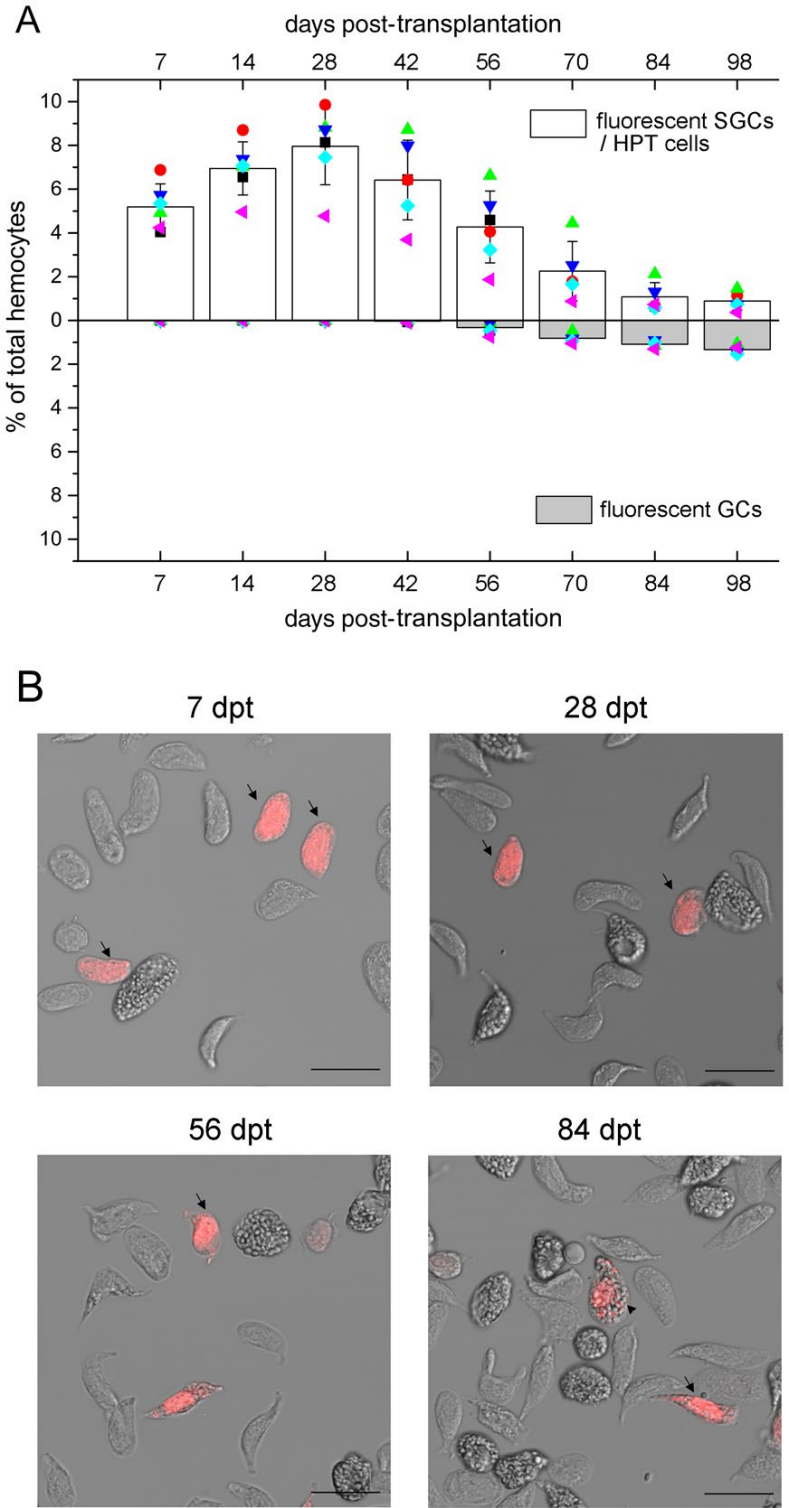
Approximately 60% of transplanted HPT cells develop into GCs. HPT cells were isolated from the donor animals, labeled with a nontoxic fluorescence cell tracer (red) and transplanted into the recipients. Hemocytes were taken from the recipients and analyzed at different time points. The percentages of fluorescent GCs and SGCs/HPT cells in the total hemocytes were determined by flow cytometry and fluorescence microscopy. Over 10,000 cells were analyzed for each sample (**A**). Five animals were used in each group. Data from different individuals are indicated by different symbols. Images of transplanted cells (red) in the samples collected at various time points are shown (**B**). Fluorescent SGCs/HPT cells are indicated with arrows, and fluorescent GCs are indicated with arrowheads. Bar, 25 μm.

Incorporation of EdU into newly synthesized DNA may have a negative effect on the cells. To monitor the development of newly produced hemocytes for a longer time, HPT cells were isolated, labeled with a nontoxic fluorescence cell tracer, and transplanted into healthy crayfish (~ 9 × 10^6^ cells per individual). Hemocytes were taken from the recipients for analysis over time, and the percentages of fluorescent cells, as well as the percentages of fluorescent GCs in the circulating hemocytes, were monitored by flow cytometry and fluorescence microscopy. Notably, it was difficult to distinguish HPT cells and SGCs using these two methods. The percentage of fluorescence-labeled cells in the circulation was relatively low at the beginning (~ 5%, varied among individuals), gradually increased, peaked at 28 dpt (~ 8%, varied among individuals), and then dropped from 42 dpt (Fig. 3). The first fluorescent GCs normally appeared at 42–56 dpt, and ~ 60% of the labeled cells developed into GCs at 3.3 months (98 days), suggesting that most cells in the HPT had a GC fate. Although the transplanted cells could be observed in the circulation for more than four months, the fluorescent signal in the cells decreased with time. We observed a shift in fluorescent populations from high to low intensity in the flow cytometry analysis (data not shown). Therefore, the drop in the number of labeled cells from 42 dpt might largely be due to the reduction of the dye in cells.

### Transplanted hemocytes develop along the GC lineage

**Figure 4:**
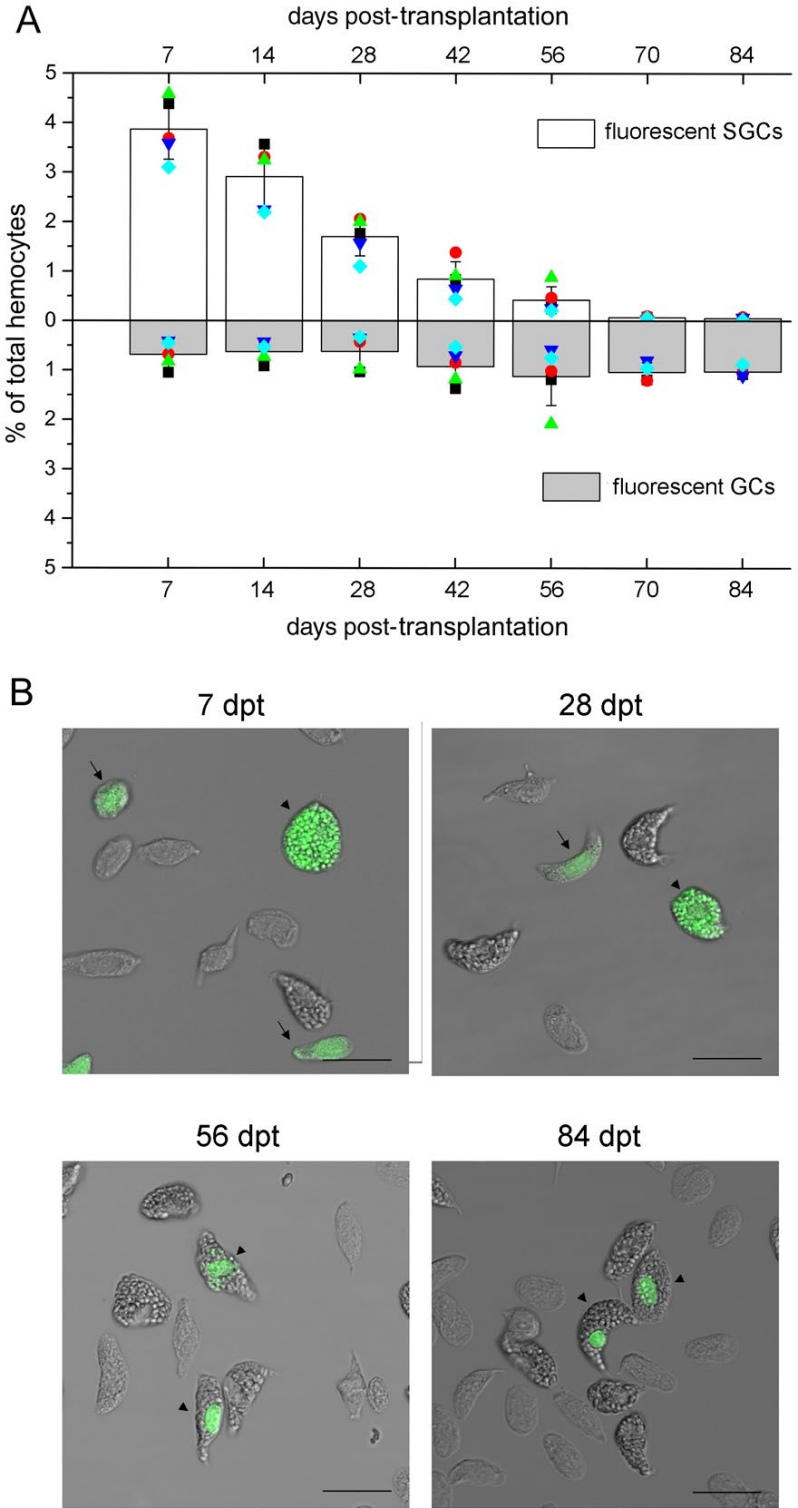
Almost all transplanted hemocytes develop into GCs. Hemocytes were isolated from donor crayfish, labeled with a nontoxic fluorescence cell tracer (green) and transplanted into recipients. Hemocytes were taken from the recipients and analyzed at different time points. The percentages of fluorescent GCs and SGCs in the total hemocytes were determined by flow cytometry and fluorescence microscopy. Over 10,000 cells were analyzed for each sample (**A**). Five animals were used in each group. Data from different individuals are indicated by different symbols. Images of transplanted cells are shown (**B**). Fluorescent SGCs are indicated with arrows, and fluorescent GCs are indicated with arrowheads. Bar, 25 μm.

Because it was difficult to investigate transplanted HPT cells for a longer time, we tracked the ongoing development of hemocytes in the circulation by total hemocyte transplantation. When similar amounts of hemocytes were allotransplanted, fewer foreign cells colonized the recipients than HPT cells. Normally, the transplanted hemocytes represented ~ 4.5% of the circulating hemocytes on day 7, when they were transplanted at a dose of 1 × 10^7^ cells per individual (Fig. 4). In contrast to transplanted HPT cells, the number of transplanted hemocytes decreased rapidly over time. The reduction was not due to the loss of fluorescent labels, since the signal in most cells was bright and no significant decline in the fluorescence intensity was observed. Mature GCs were more difficult to survive in recipients, since the GC percentage of transplanted cells was significantly reduced after transplantation (GC percentage of the source cells: ~ 26%; GC percentage 7 dpt: ~ 15%). A similar case was observed when autologous hemocytes were transplanted (data not shown). This reduction might be due to immune reactions targeting transplanted cells, regardless of whether they were allogenic or autologous. Foreign hemocytes could be observed in the circulation for more than three months. There was an obvious increase in the percentage of fluorescent GCs at ~ 28 dpt, when ~ 25% of the allogenic hemocytes were GCs. Since 70 dpt, GCs have accounted for over 94% of the allogenic cells remaining in the circulation. The percentage reached 100% at 84 dpt in some animals. The time required for maturation of the transplanted hemocytes depended on the cells from donors and the physical status of recipients. These data suggested that almost all of the hemocytes belonged to the GC lineage.

Interestingly, when hemocytes were labeled with the cell tracer CFDA SE, the fluorescent signal in GCs was much stronger than the fluorescent signal in SGCs, especially in the cytoplasmic granules. After injection into the recipients, the fluorescent signal in the cytoplasm gradually decreased, whereas the signal in the granules of GCs and in the nucleus of both types of cells remained strong (Figs. 4B and S2). This might because the half-lives of nuclear proteins and the proteins inside cytoplasmic granules were much longer than the half-lives of free proteins in the cytoplasm. The granules in SGCs were small; thus, the fluorescent signal in these granules could not be distinguished from the signal in the cytoplasm initially. In the first 2 weeks post-transplantation, most of the fluorescent GCs contained large amounts of fluorescent granules (Figs. 4B, S2A,B), suggesting that they were original GCs from the source. The percentage of this type of fluorescent GCs decreased with time, while the percentage of GCs containing few fluorescent granules (Figs. 4B, S2C,D) and GCs with only a nuclear fluorescent signal (Figs. 4B, S2E,F) increased. Finally, at ~ 84 dpt, > 90% of the labeled GCs bore only a nuclear fluorescent signal (Figs. 4B, S2E,F), suggesting that they were derived from less differentiated cells of the donors.

### Transplanted SGCs develop along the GC lineage

**Figure 5:**
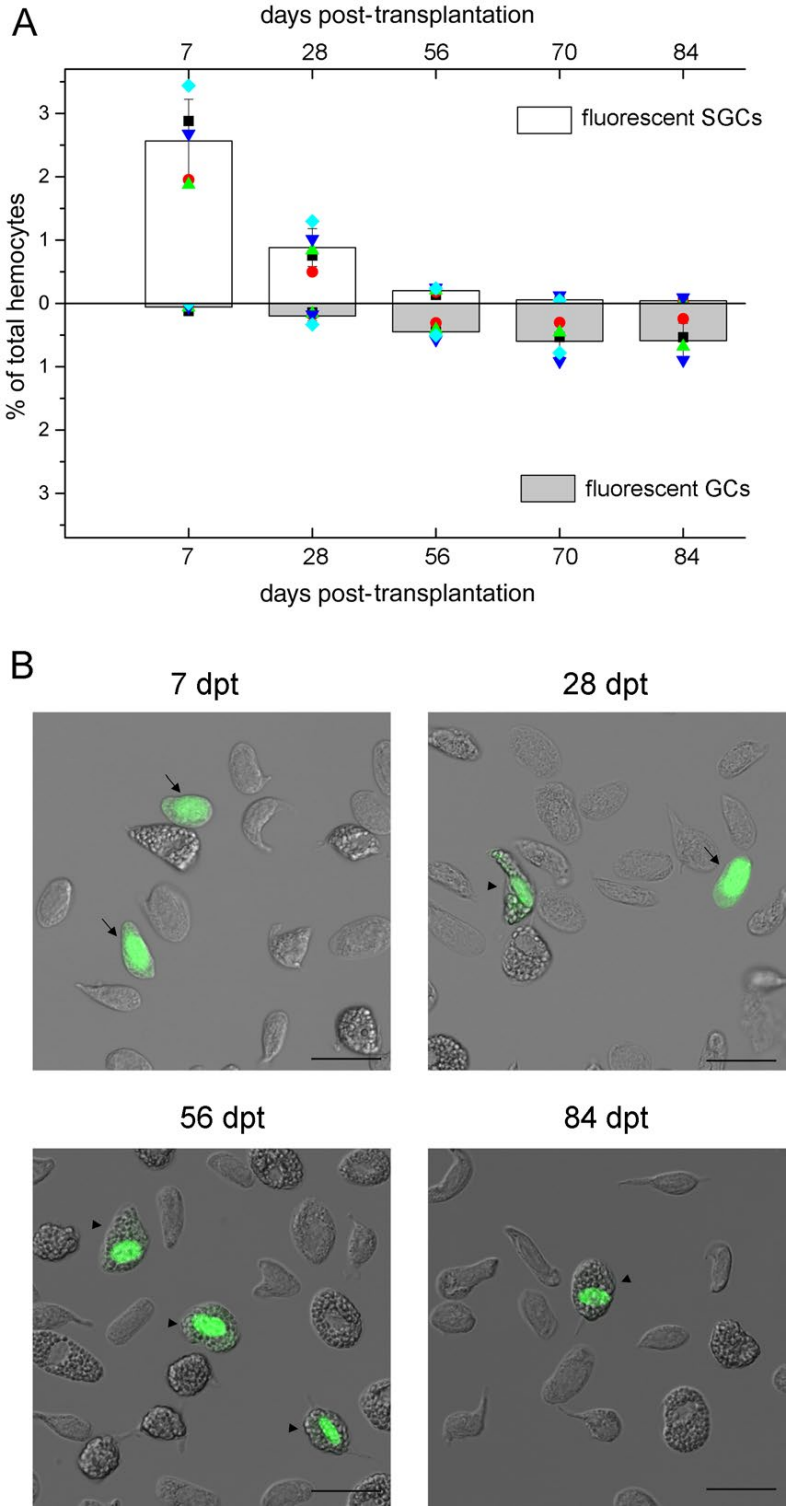
Almost all transplanted SGCs develop into GCs. The purified SGCs were labeled with a nontoxic fluorescence cell tracer (green) and transplanted into the recipients. Hemocytes were taken from the recipients and analyzed at different time points. The percentages of fluorescent GCs and SGCs in the total hemocytes were determined by flow cytometry and fluorescence microscopy, and over 10,000 cells were analyzed for each sample (**A**). Five animals were used in each group. Data from different individuals are indicated by different symbols. Images of transplanted cells are shown (**B**). Fluorescent SGCs are indicated with arrows, and fluorescent GCs are indicated with arrowheads. Bar, 25 μm.

To confirm that most of the SGCs finally developed into GCs, purified SGCs (> 98% purity determined by flow cytometry and microscopy analysis, Fig. S3) were labeled and allotransplanted. Samples were collected at 7, 28, 56, 70, and 84 dpt for analysis (Fig. 5). As we observed in total hemocyte transplantation, only a small portion of foreign cells survived, and the percentage of allogenic cells continuously decreased in the circulation. The development of allogenic SGCs into GCs was first observed at 28 dpt, and then > 94% of the allogenic SGCs converted into GCs at 84 dpt. The timing was consistent with the timing of the total hemocyte transplantation. In this experiment, most of the labeled GCs formed in the first month contained few fluorescent cytoplasmic granules, suggesting that they were derived from SGCs with few granules. From the second month, most of the labeled GCs contained fluorescent signals in the nucleus only (Fig. 5B), suggesting that they were derived from SGCs with no granules. These results supported the findings in total hemocyte transplantation.

We also transplanted purified GCs (> 97% purity, Fig. S3) into crayfish (4 × 10^5^ cells/individual). Very few GCs successfully colonized the recipients, accounting for < 0.5% of the total circulating hemocytes. Some of the GCs could survive for ~ 2 months. In our experiment, fewer than 100 allogenic cells could be found in the hemolymph sample we took at each time point. Most of the allogenic cells remained as GCs for 2 months (Table S3). Occasionally, 1 ~ 2 allogenic SGCs could be seen in the first week but not in the late time points, which might be due to degranulation of the transplanted GCs or the contamination of SGCs in the purified GCs. These results indicated that GCs did not develop into SGCs.

### The development of hemocytes can be divided into three stages

**Figure 6:**
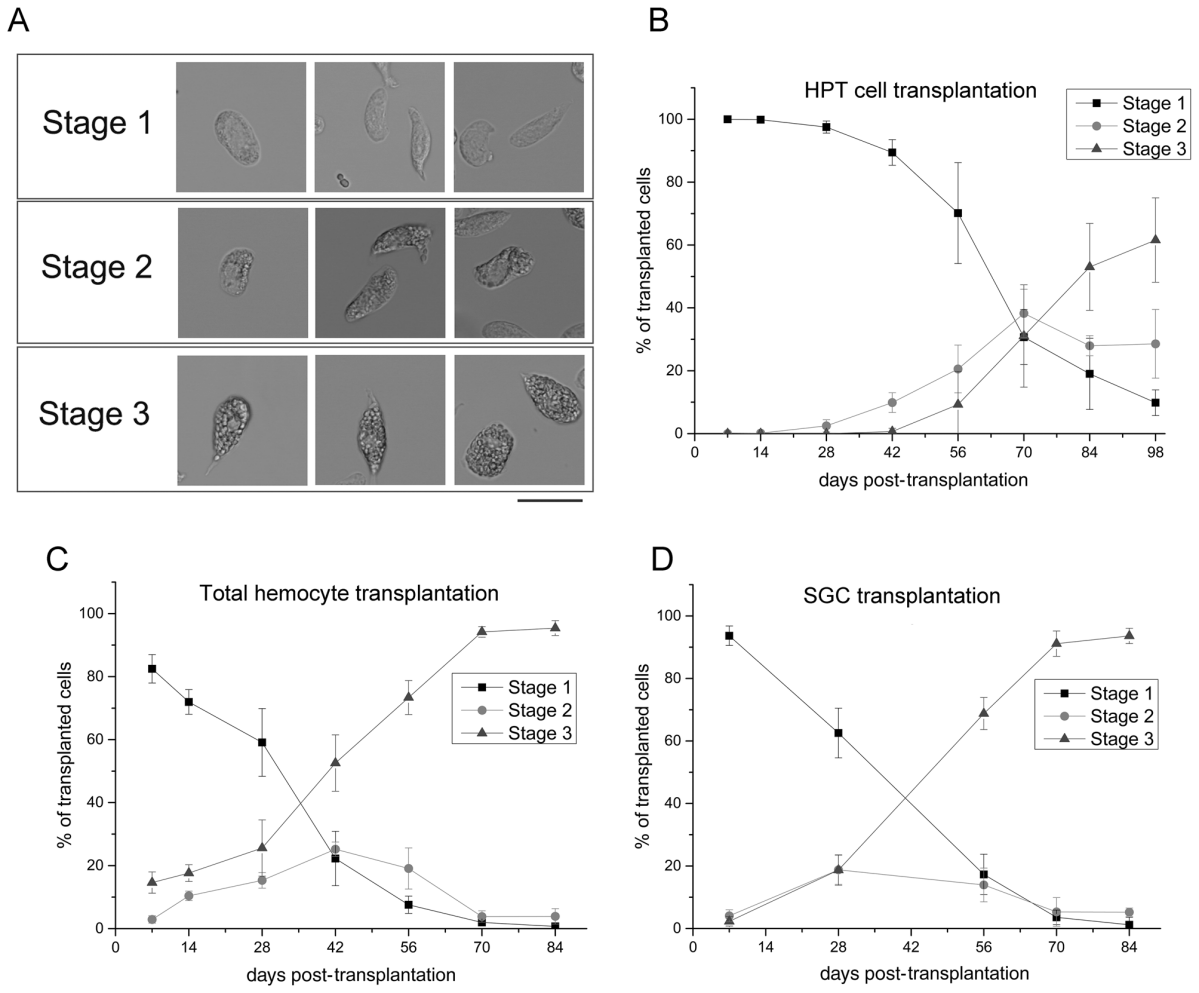
The development of transplanted cells can be divided into three stages. In the transplantation experiments, the differentiation and maturation of hemocytes could be roughly separated into three morphological stages (Stage-1, -2, -3) based on light microscopy observations (**A**). Stage-1, early hemocytes with no/some small granules; Stage-2, cells with some medium size granules; Stage-3, mature cells filled with many large granules. The changes in the proportions of cells in these three stages during the developmental process are shown: (**B**) HPT cell transplantation; (**C**) total hemocyte transplantation; (**D**) SGC transplantation. Bar, 25 μm.

Traditionally, circulating hemocytes in crayfish are classified into GCs and SGCs (disregard rare HCs). In the results described above, we adopted this nomenclature, and only cells with many large cytoplasmic granules were considered GCs, while the others were considered SGCs. However, in the transplantation experiments, we did identify a group of SGCs that contained some medium-sized granules. This group of SGCs seemed to be an intermediate form, which did not fit the typical features of SGCs or GCs very well. Thus, the development of transplanted hemocytes was refined into three morphological stages based on light microscopy observations. Stage-1, early hemocytes with no/some small granules, previously classified as SGCs; Stage-2, cells with some medium-sized granules, previously classified as SGCs; Stage-3, mature cells filled with many large granules, previously classified as GCs (Fig. 6A). In untransplanted animals, Stage-2 cells normally accounted for ~ 10% of the circulating cells (data not shown), which was less than the percentage of Stage-1 and Stage-3 cells. The distribution of transplanted cells belonging to different stages is summarized for all the transplantation experiments in Fig. 6B–D. Basically, the percentage of Stage-1 cells decreased with time; the percentage of Stage-2 cells first increased and then decreased, whereas the percentage of Stage-3 cells continuously increased. In HPT cell transplantation, Stage-2 cells emerged prior to Stage-3 cells. In addition, the maximum content of Stage-2 cells was always below 50% in all the experiments. These results suggested the sequential development of newly released hemocytes and indicated that Stage-2 was a transition stage of hemocyte development.

### The expression of marker genes confirms the development of hemocytes along the GC lineage

**Figure 7:**
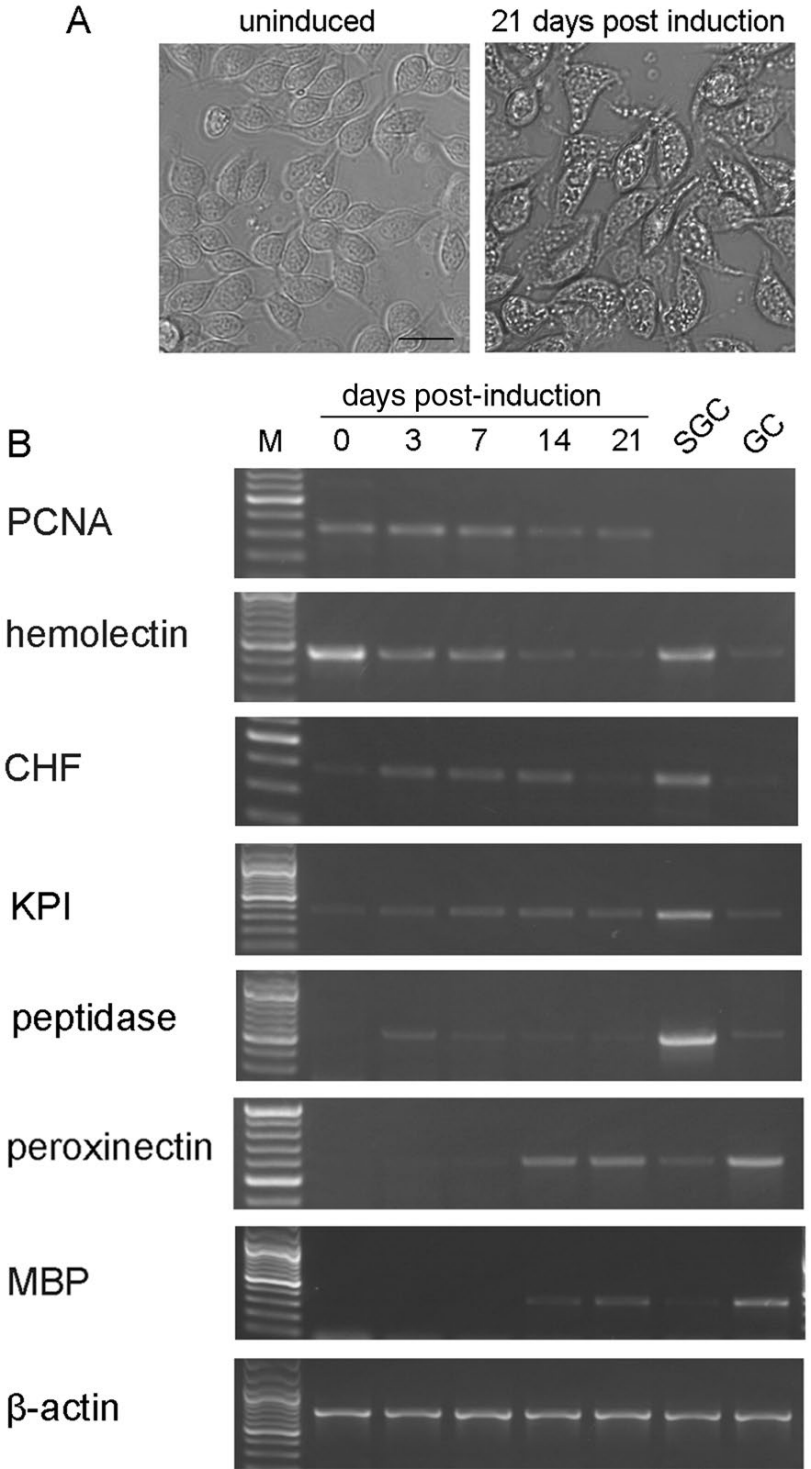
The expression of marker genes indicates a sequential development from HPT cells to SGCs and finally to GCs. The differentiation of GCs from HPT cells was induced in vitro by the addition of crayfish muscle extract. (**A**) Images of uninduced HPT cells and cells induced for 21 days. Bar, 20 μm. (**B**) RNA was extracted from the cells at different times post-induction, and the expression of marker genes was analyzed by RT-PCR. Purified SGCs and GCs were used as the controls. There was a sequential change in the gene expression pattern from undifferentiated HPT cells to SGCs and finally to GCs. The genes analyzed were PCNA (proliferating cell nuclear antigen, HPT cell marker); hemolectin (expressed in HPT cells and SGCs); CHF (crustacean hematopoietic factor, expressed in HPT cells and SGCs); KPI (SGC specific Kazal proteinase inhibitor, SGC marker); peptidase (SGC marker); peroxinectin (GC marker); and MBP (mannose-binding protein, GC marker). β-actin was used as an internal control.

The differentiation of GCs from HPT cells can be induced in vitro^40^. We further analyzed the expression of hemocyte marker genes during GC differentiation (Fig. 7). These genes included proliferating cell nuclear antigen (PCNA, HPT cell marker)^22^, SGC specific Kazal proteinase inhibitor (KPI, SGC marker)^22,46^, peptidase (SGC marker, unpublished data), hemolectin (expressed in HPT cells and SGCs)^46^, CHF (expressed in HPT cells and SGCs)^28,46^, mannose-binding protein (MBP, GC marker, unpublished data), and peroxinectin (GC marker)^46^. Purified SGCs and GCs were used as the controls. As shown in Fig. 7A, most cells developed into GCs (with numerous cytoplasmic granules) within 21 days of induction. The expression of PCNA was relatively high in uninduced HPT cells and early-differentiated (3–7 days post-induction) cells and gradually reduced with the progression of differentiation. The expression of the GC markers MBP and peroxinectin was not detectable until 14 days post-induction. The SGC markers were not very specific. They were highly expressed in SGCs but also expressed in GC (KPI, peptidase) or HPT cells (KPI, hemolectin, and CHF) at different levels. The expression of hemolectin, CHF, and peptidase was detected in undifferentiated cells and cells induced for 3–14 days with a gradual reduction, but not in late-differentiated cells (21 days post-induction). KPI was detected in all the cells we analyzed. Despite the nonspecificity of some marker genes, the change in the gene expression pattern from undifferentiated HPT cells to SGCs and finally to GCs was clearly observed in the process of GC differentiation (Figs. 7B and S4).

### Hemocytes of different developmental stages are capable of phagocytosis

To determine whether hemocytes of different developmental stages are functional immune cells, we performed an in vivo phagocytosis assay. Animals were injected with fluorescence microspheres, and hemolymph samples were analyzed at 4 hpi. We found that cells belonging to all three stages were capable of phagocytosis (Fig. S5). In our experiment, the overall phagocytic rate of circulating hemocytes was normally < 7%. Stage-1, -2, and -3 cells accounted for approximately 65%, 15%, and 20% of the phagocytic population, respectively. Notably, for each stage, only a small portion of cells were phagocytic cells, even though an excessive number of microspheres were used. These results suggested that the developing cells in circulation were already functional immune cells, and there were functionally different subpopulations.

## Discussion

### Crayfish hemocytes develop along the GC lineage

The aim of this study was to explore the process of hemocyte development in crustaceans using crayfish as a model animal. SGCs and GCs are the dominant types of circulating hemocytes in crayfish^14,22,23^ and have long been considered mature cells. A hypothetical model for bilineage hemocyte differentiation has been proposed^16^. Basically, pluripotent progenitor cells give rise to common precursors for SGCs and GCs, which then differentiate along either the SGC or GC lineage. This model is supported by some morphological and molecular evidence^30,46^, but it is far from complete. Our findings in the current study challenge this model. We propose that crayfish hemocytes develop along a single lineage, the GC lineage. The long-known SGCs and GCs may represent hemocytes of different developmental stages rather than two types of fully differentiated cells. Rare HCs are prohemocytes occasionally released from HPT^14^.

First, according to research in other animals, if blood cells differentiate in a multilineage manner, the production of different types of cells (with labels) will soon be seen after EdU injection or hematopoietic stem cell/progenitor cell transplantation^9,11,47^. However, this is not the case in crayfish. There are no mature GCs in the HPT of crayfish^30,40^. EdU-incorporated hemocytes are released into the circulation in the form of SGCs and take approximately one month to mature. Consistently, the transplanted HPT cells take 1.5 ~ 2 months to become GCs. Therefore, crayfish hemocytes may not differentiate in a multilineage manner, and SGCs may be immature GCs.

Second, almost all the transplanted hemocytes finally developed into GCs. According to in vitro^40^ and in vivo differentiation experiments, most of the progenitors in HPT have a GC fate. When total hemocytes or purified SGCs are transplanted, 94 ~ 100% of the allogenic cells finally develop into GCs. GCs are the main senescent cells in the circulation, representing the terminal stage of differentiation^18,39,46^. GCs cannot reversely convert into SGCs. These results strongly suggest that all crayfish hemocytes belong to the GC lineage.

Third, molecular and morphological evidence supports the single lineage development of hemocytes. During GC differentiation, there is a sequential change in the gene expression pattern from undifferentiated HPT cells to SGCs and finally to GCs, indicating a differentiation procedure from progenitor cells through SGCs to GCs. Overlap expression of SGC markers in HPT cells or GCs further verifies SGC as an intermediate form. This result is in agreement with a global proteomic analysis of the hematopoietic lineages in crayfish, which showed that SGCs are similar to but less differentiated than GCs^46^. Moreover, we refined the development of hemocytes into three successive stages. In the transplantation experiments, a sequential development of allogenic cells from early Stage-1 to transitional Stage-2 and finally to mature Stage-3 was observed.

Finally, although the hemocytes show similar morphological changes during development, they do not need to be identical. Subpopulations with different functions are very likely to exist. Cells of all three developmental stages are capable of phagocytosis. However, for each stage, only a small portion of the cells are phagocytic. Thus, there may be a phagocytic subpopulation that becomes functional soon after entering the circulation. The heterogeneity of cells in the same morphological group is supported by previous findings showing their difference in staining properties^24–26^_,_ gene expression patterns^27,28^, or immune function^23,29^.

Altogether, we conclude that crayfish hemocytes develop along a single lineage, the GC lineage, which may contain functionally different subpopulations. Although replenishment of blood cells is important for all multicellular animals, hematopoiesis has only been studied in a small number of phyla. Based on knowledge from vertebrates^48^ and a few invertebrates, such as insects^6,49^, echinoderms^50^, and tunicates^9^, blood cells normally differentiate in a multilineage manner. HSCs give rise to common progenitor cells, which differentiate into lineage-committed cells, and then develop into various types of blood cells. The idea of hemocyte development along a single lineage has been proposed in some early studies of crustaceans^18,38,39^ and mollusks^51,52^ but with limited evidence. Here, we provide convincing evidence for a single lineage schema for crayfish hemocyte development. Further study is needed to determine whether it is a similar case in other crustaceans or even other phyla.

Despite the diverse and confusing nomenclature, invertebrate blood cells can be classified into four major types: prohemocytes, plasmatocytes/HCs, granular hemocytes, and eleocytes^1^. Morphologically and functionally, crayfish SGCs correspond to plasmatocytes/HCs (resembling monocytes/macrophages in vertebrates), which are phagocytic cells with relatively transparent cytoplasm^1,53^. GCs correspond to granular hemocytes (resembling granulocytes in vertebrates), which produce cytotoxic materials and are capable of phagocytosis^1,54–56^. The rare HCs in crayfish are best compared to prohemocytes^1,14^. Phagocytes and granular hemocytes are ancient types of immune cells. Phagocytes already develop in protozoa, and granulocyte-like cells are found in the lower chordates (cephalochordate and urochordate) and other invertebrates, including echinoderms, urochordates, crustaceans, mollusks, annelids, and insects^1,13,14,49^. Additionally, we show that crayfish SGCs further develop into GCs. The differentiation of one type of hemocyte into another has been observed in other animals. Drosophila plasmatocytes can further develop into lamellocytes or crystal cells^7^. Mammalian monocytes can exit the bloodstream and develop into tissue-resident macrophagocytes or dendritic cells^1,53^. Conversion of one type of immune cell into another when needed may be a strategy that evolved to increase the flexibility and effectivity of the immune system.

### Crayfish hemocytes are long-lived cells that develop at a relatively slow speed

The timing for hemocyte development was estimated based on our data. First, over 95% of the cells in HPT become EdU-incorporated on day 4 post EdU injection, and EdU-incorporated hemocytes appear in circulation on day 3 to day 4. Thus, immature hemocytes derived from proliferating progenitors need to differentiate in HPT for 3 ~ 4 days before being released. Second, crayfish HPT lobules contain no GCs^30,40^, and the first EdU-labeled GCs in circulation appear one month later. Thus, new hemocytes released to circulation may take at least a month to mature. Notably, the time required for transplanted HPT cells to mature (42–56 days) is longer than the time required for EdU-incorporated cells to mature. The cells in HPT may be more primitive and they may encounter certain rejection effects when transplanted. Third, the time required for > 94% of transplanted hemocytes to develop into GCs is ~ 3 months, whereas the first GCs mature in a month. Why is there such a long time-span? The cell type composition in circulation is under strict control and does not respond strongly to circadian rhythm or stimuli such as the injection of beads and buffer saline^23^. After receiving hemocyte transplantation, the body may still try to maintain this balance. Thus, the transplanted cells develop into GCs successively but not simultaneously. The more preliminary cells may mature at a slower speed. The mature GCs can live for as long as 2 months. Thus, the hemocytes of crayfish can be very long-lived. Similarly, there are long-lived hemocytes in sea urchin^11^. This is very different from monocytes and granulocytes in vertebrates, which survive in the bloodstream only for several days^56,57^. Long-lived immune cells may provide invertebrates with a more powerful and effective innate immune system. Finally, hemocytes in all three developmental stages are capable of phagocytosis, suggesting that immature hemocytes become functional soon after their release. Taken together, the immature hemocytes derived from proliferating progenitors differentiate in HPT for 3 ~ 4 days and continue to develop in the circulation for at least a month.

### Approximately 1.8% of circulating hemocytes are replaced every day

We also estimated the hemocyte replacement rate by EdU uptake. The increase in EdU-incorporated cells in the HPT was roughly linear from day 1 to day 3 post injection. Correspondingly, the portion of EdU-incorporated hemocytes in circulation increased in a linear way from day 3 to day 6. If HPT contains a relatively stable number of cells, it produces ~ 15% new cells and releases ~ 15% of its cells to the circulation every day. Accordingly, ~ 1.8% of the circulating hemocytes are replaced per day, and ~ 1% of the circulating hemocytes are dead. These are important parameters for the immune system that have not been reported before.

### Over 20% of the cells in HPT are proliferating progenitors

According to research in other animals, there are basically three types of cells in the HPT: relatively quiescent hematopoietic stem cells (HSCs), actively proliferating progenitor cells, and lineage-committed blood cells in various differentiation stages^6,58,59^. In crayfish, ~ 20% of cells in the HPT incorporated EdU at 2 hpi. Since a 2 h time window is normally not enough for cells to complete the cell cycle, we deduce that > 20% of the cells in HPT are active proliferating progenitors. In the EdU uptake assay, there were always < 5% unincorporated cells. These unincorporated cells might be circulating hemocytes infiltrating the tissue or quiescent cells that did not proliferate during our experimental procedure. However, there is no direct evidence for the presence of quiescent HSCs in crustaceans.

## Conclusions

**Figure 8:**
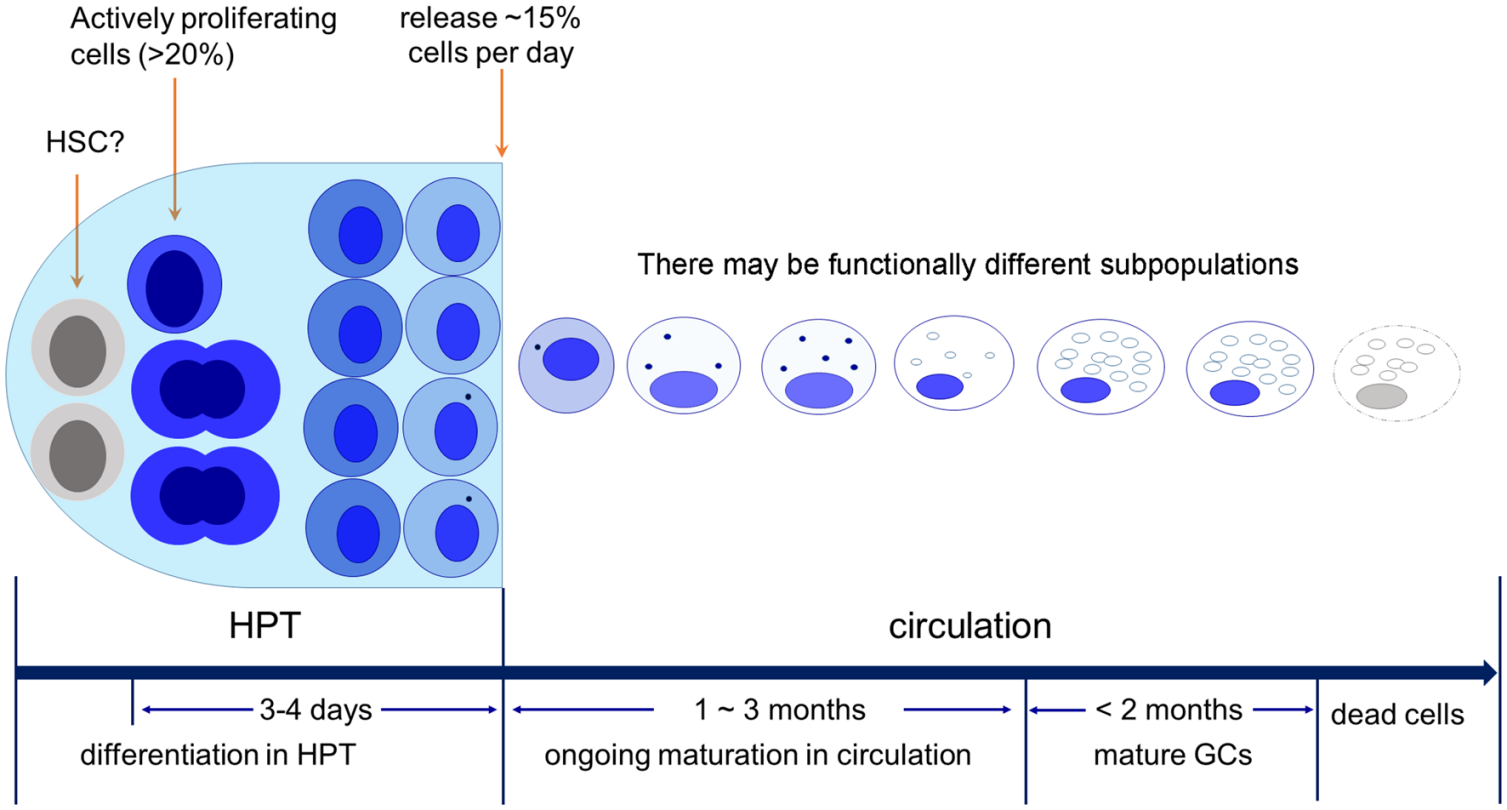
A hypothetical model for crayfish hemocyte development. Crayfish hemocytes develop along a single lineage, the GC lineage. Active proliferating progenitor cells represent > 20% of the population in HPT. The hemocyte precursors produced by progenitor cells differentiate in HPT for 3–4 days. Immature hemocytes are released into the circulation in the form of SGCs, which continue to develop in circulation for at least one month before maturity. GC represents the terminal stage of hemocyte development. Because the portion of GCs in circulation is relatively stable, the time required for cell maturation may extend to ~ 3 months. Mature cells can survive for as long as 2 months. Although hemocytes show similar morphological changes during development, they do not need to be identical. Subpopulations with different functions are very likely to exist.

In this study, we explored hemocyte development in crayfish by tracking newly produced hemocytes and transplanted cells. We demonstrate that nearly all circulating hemocytes finally differentiate into GCs. The long-known SGCs and GCs may represent hemocytes at different developmental stages. The timing of development, as well as the life span and replacement rate of circulating hemocytes, are estimated. A hypothetical model for hemocyte development is shown in Fig. 8. In this model, the hemocyte precursors produced by progenitor cells differentiate in HPT for 3 ~ 4 days. Immature hemocytes are released into the circulation in the form of SGCs, which continue to develop in circulation for at least one month before maturity. GC represents the terminal stage of hemocyte development. Because the portion of GCs in circulation is relatively stable, the time required for cell maturation may extend to ~ 3 months. Mature cells can survive for as long as 2 months. Although hemocytes show similar morphological changes during development, they do not need to be identical. Subpopulations with different functions are very likely to exist. These findings may not only reshape our understanding of immune cells and their development in crustaceans, but also shed light on the evolution of hematopoiesis in the animal kingdom.

## Supplementary Information

Supplementary Information.

## Abbreviations

GC: Granular cell
SGC: Semigranular cell
HC: Hyaline cell
HPT: Hematopoietic tissue
PI: Propidium iodide
EdU: 5-Ethynyl-2’-deoxyuridine
dpi: Days post-injection
hpi: Hours post-injection
dpt: Days post-transplantation
AST1: Astakine 1
CHF: Crustacean hematopoietic factor
PCNA: Proliferating cell nuclear antigen
KPI: Semigranular cell specific Kazal proteinase inhibitor
MBP: Mannose-binding protein
HSC: Hematopoietic stem cell
