# Supplementary material for: Crayfish hemocytes develop along the granular cell lineage
Source: Sci Rep. 2021 Jun 23;11:13099. doi: 10.1038/s41598-021-92473-9 (PMC8222279; doi:10.1038/s41598-021-92473-9)
Supplement: Supplementary file 1 — Supplementary Information. [file 41598_2021_92473_MOESM1_ESM.pdf]

Crayfish hemocytes develop along the granular cell lineage

Fang LI[1,2]*, Zaichao ZHENG[1], Hongyu LI[1], Rongrong FU[1], Limei XU[1,2], Feng YANG[1,2]*

[1] Key Laboratory of Marine Genetic Resources, Third Institute of Oceanography, Ministry of Natural Resources, Xiamen 361005, China.

[2] Fujian Key Laboratory of Marine Genetic Resources, Xiamen 361005, China.

* Corresponding authors: Third Institute of Oceanography, Ministry of Natural Resources, 184# Daxue Road, Xiamen 361005, PR China. Tel.: +86 592 2195015. E-mail addresses: lifang@tio.org.cn (for F. Li), yangfeng@tio.org.cn (for F. Yang)

Supplementary materials

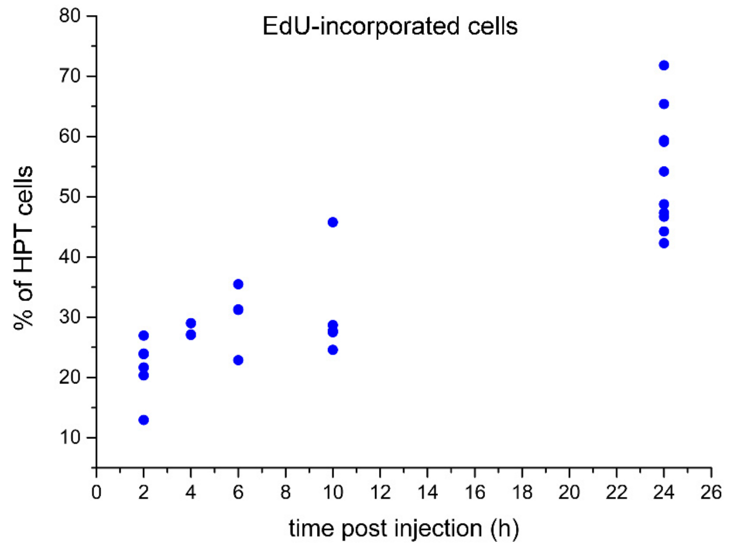

Fig. S1 EdU incorporation in HPT cells within 24 h

Each crayfish was injected with one dose of EdU. HPT cells were collected from each animal at different time points until 24 hpi. After fixation, cells were stained and analyzed by flow cytometry. The percentages of EdU-incorporated cells in HPT are plotted with time. More than 4 animals were analyzed at each time point.

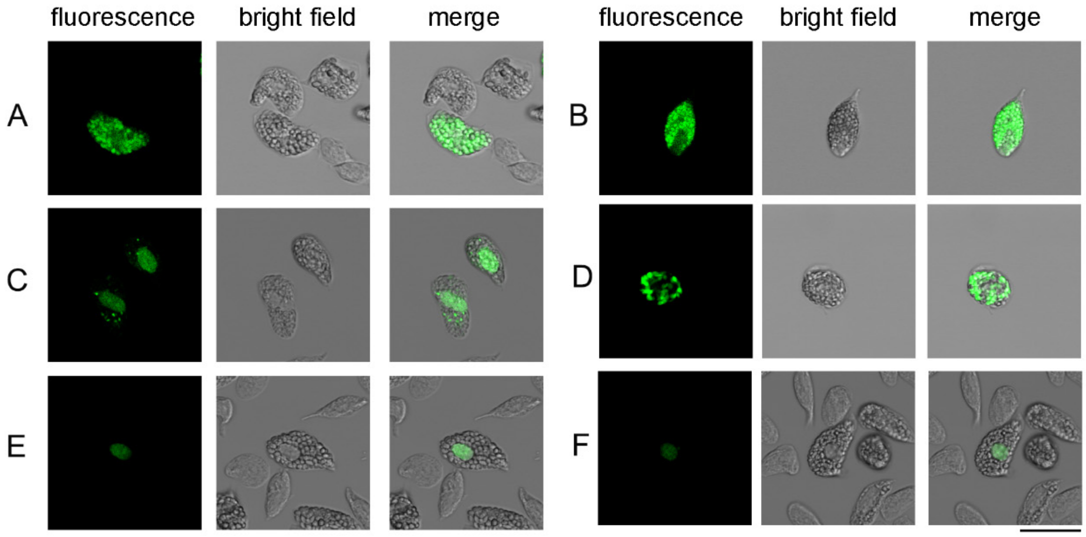

Fig. S2 Images of allogenic GCs in the recipients

Hemocytes were labeled with the fluorescent dye and transplanted to the recipients. The transplanted cells (green) were observed by fluorescent microscopy over time. (A, B) GCs filled with many fluorescent cytoplasmic granules at 7 dpt. (C, D) GCs contain a few fluorescent cytoplasmic granules at 42 dpt. (E and F) GCs with fluorescent signal in the nucleus only at 84 dpt. Bar, 25 μm.

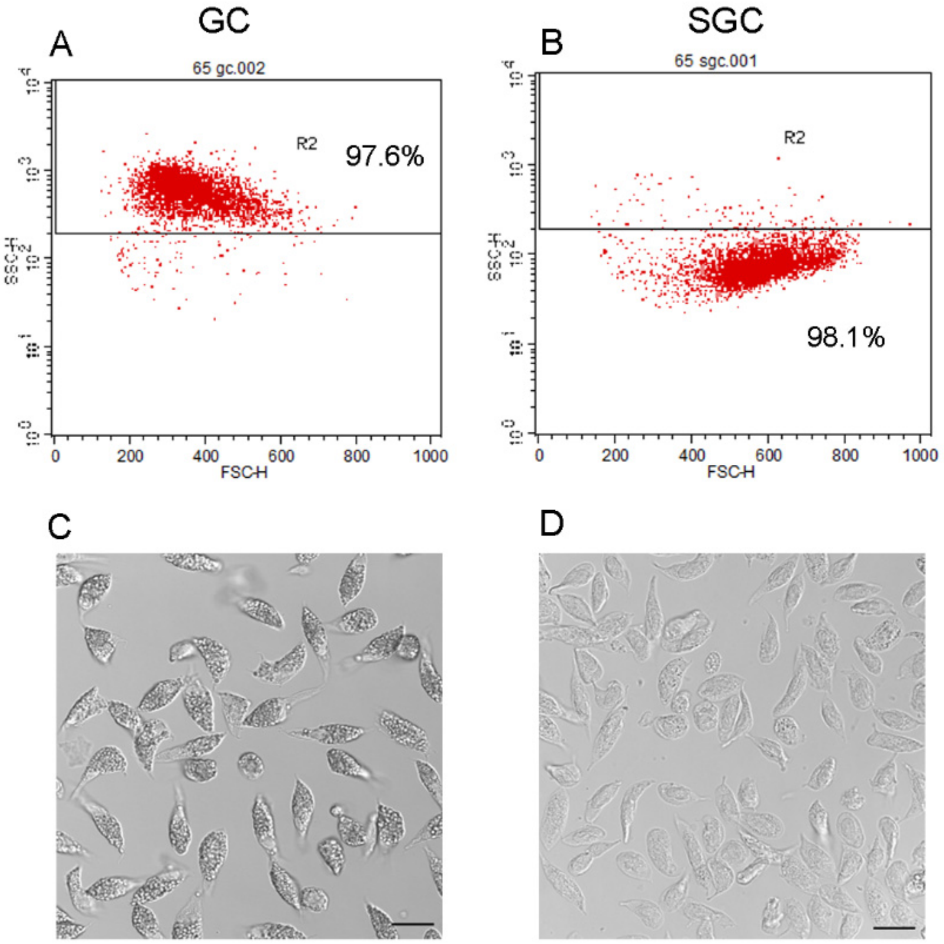

Fig. S3 SGCs and GCs used in the transplantation experiment are of high purity

Hemocytes were collected from crayfish and purified by Percoll density gradient. The purity of GCs (A) and SGCs (B) was analyzed by flow cytometry with FSC-SSC scatter plots. The population in the upper region of each plot are GCs, while the population in the lower region are SGCs. The cells were further observed under a microscope: C. GCs; D. SGCs; bar 25 μm.

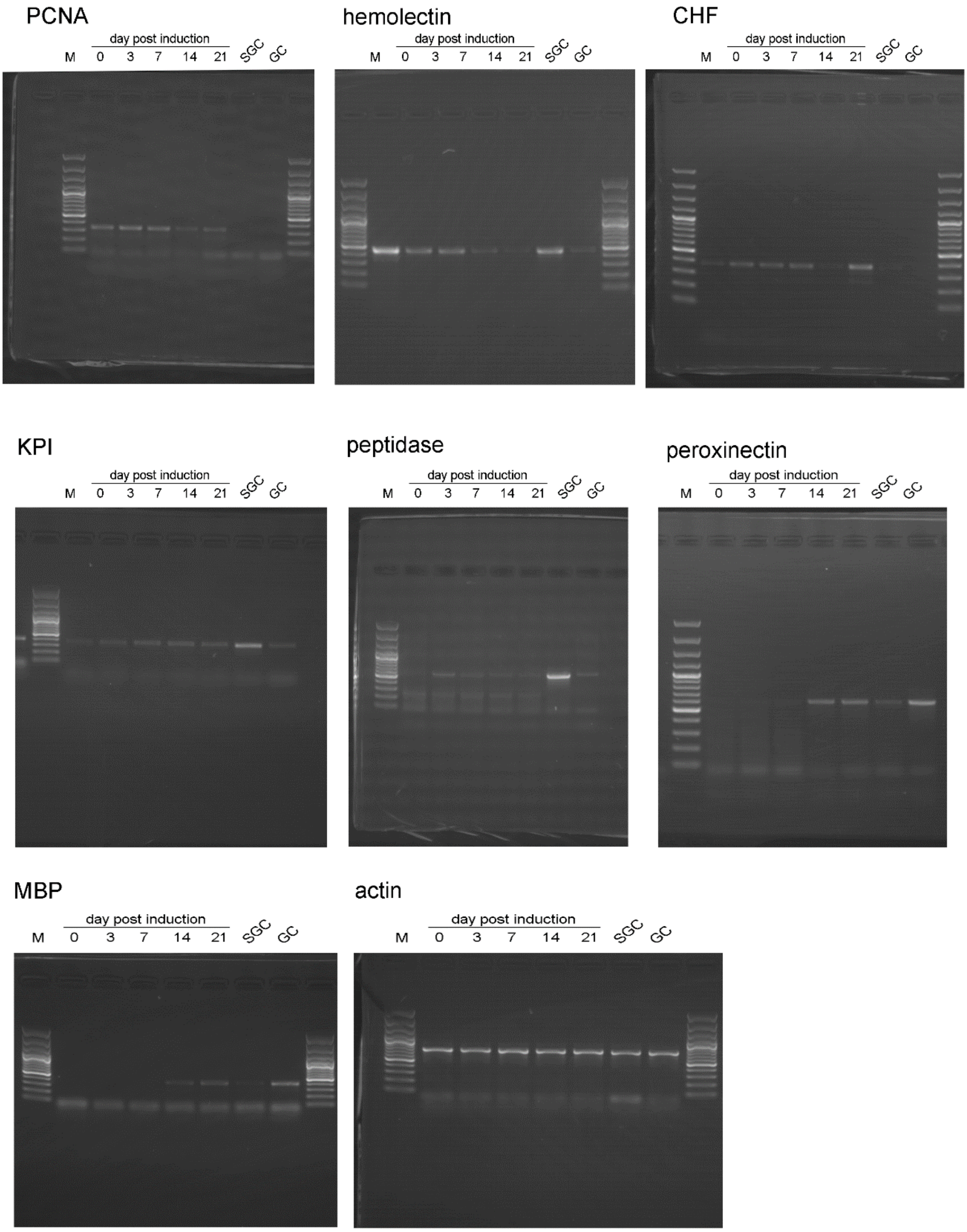

Fig. S4 Original images of the electrophoresis results in Fig. 7 "The expression of marker genes indicates the sequential development from HPT cells to SGCs and finally to GCs"

The differentiation of GCs from HPT cells was induced *in vitro* by the addition of crayfish muscle extract. RNA was extracted from the cells at different times post-induction, and the expression of marker genes was analyzed by RT-PCR. Purified SGCs and GCs were used as the controls. The genes analyzed were PCNA (proliferating cell nuclear antigen, HPT cell marker); hemolectin (expressed in HPT cells and SGCs); CHF (crustacean hematopoietic factor, expressed in HPT cells and SGCs); KPI (SGC specific Kazal proteinase inhibitor, SGC marker); peptidase (SGC marker); peroxinectin (GC marker); and MBP (mannose binding protein, GC marker). β-Actin was used as an internal control.

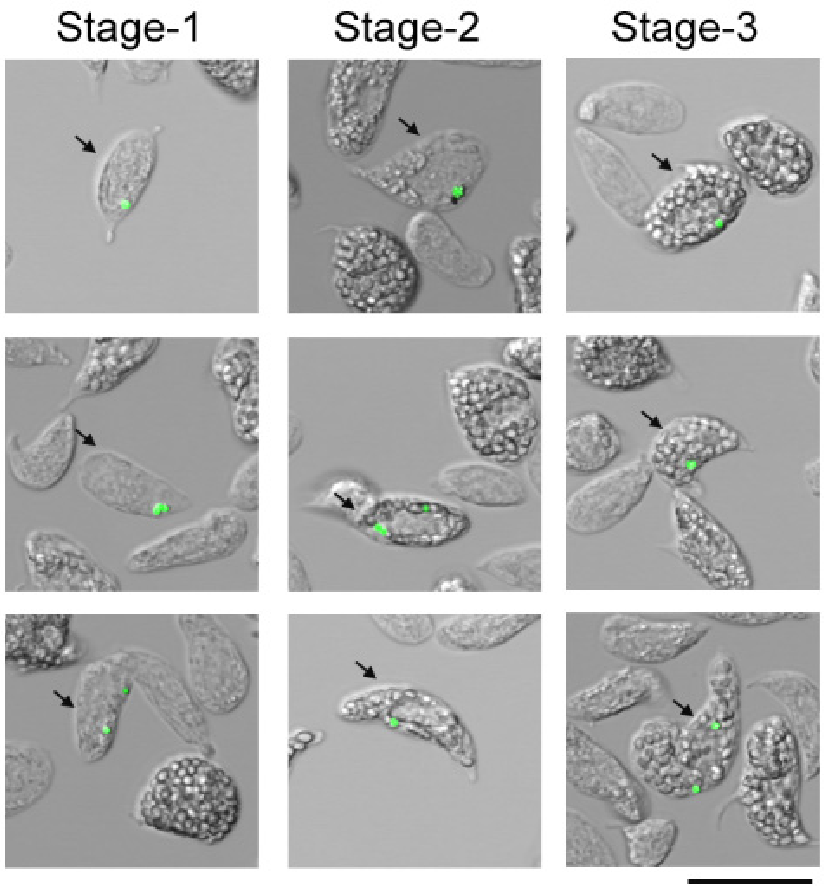

Fig. S5 Cells belonging to different developmental stages are capable of phagocytosis

Fluorescent microspheres (green) were injected into crayfish, hemocytes were collected at 4 hpi, and observed by fluorescent microscopy. Images of phagocytic cells (indicated with arrows) belong to three different stages are shown. Bar, 25 μm.

Table S1. Primers used in this study

Gene	GenBank no.	Primer	Primer sequence (5’-3’)
PCNA	MK252700	PCNA F2	TAATGGGCATGGATCTTACC
		PCNA R2	CCAATGTCACCAGCAGCAG
peroxinectin	MW358640	peroxinectin-F	ACGAGCAGCCTATCTTGACC
		peroxinectin-R	AGCTGTTGTAGGTGGCGATG
MBP	MW358645	MBP-F	CAACAAGCTAATGGTTACTGC
		MBP-R	TAAGTCTGAGTGTTGCGGTA
CHF	MW358641	CHF-F	TAACATAATGGCTCAGGTCTC
		CHF-R	AGATGTAATCGCAGTAAGTGTC
peptidase	MW358646	peptidase-F	AGTCCAGACGTGTCGAGCAT
		peptidase-R	CCAGCGAGTCCAGAGTAAGAG
KPI	MW358644	KPI-F	TGTGGAAGGTACTGTGGGTC
		KPI-R	CTGGAGATGGGTTCTTGATT
hemolectin	MW358643	hemolectin-F	CACTACCAAAACGAACATGC
		hemolectin-R	TTGGGAACCCCTACTCATAC
β-Actin	AY430093	β-actin F	GACATGGAGAAGATCTGG
		β-actin R	GCAGTGATTTCCTTCTGC

Table S2. GCs are the major senescent cells in circulation

crayfish	male/female	body weight (g)	hemocyte mortality [a]	the number of dead cells observed [b]	dead cells with intact structure [b]	dead GCs [b]	dead GCs/ dead cells with intact structure
1	male	28	0.9%	98	56	53	94.6%
2	male	35	1.8%	154	82	78	95.1%
3	male	37	1.5%	175	94	87	92.6%
4	male	41	0.4%	158	71	63	88.7%
5	male	41	0.9%	165	102	91	89.2%
6	male	42	1.0%	138	110	102	92.7%
7	male	42	0.9%	130	90	85	94.4%
8	male	43	0.6%	143	70	63	90.0%
9	male	46	1.3%	132	97	93	95.9%
10	male	57	0.6%	180	82	77	93.9%
11	female	34	0.7%	202	108	99	91.7%
12	female	37	1.2%	136	104	103	99.0%
13	female	37	0.6%	125	95	92	96.8%
14	female	40	0.8%	146	71	66	93.0%
15	female	42	0.8%	136	93	86	92.5%
16	female	44	1.0%	164	108	104	96.3%
17	female	46	1.0%	139	104	102	98.1%
18	female	50	1.3%	165	67	66	98.5%
19	female	50	0.7%	138	80	74	92.5%
20	female	60	1.2%	208	142	136	95.8%
**Average**			**1.0%**				**94.1%**
**SD**			**0.3%**				**2.9%**

[a.] Flow cytometry data; [b.] data from fluorescence microscopy analysis

Table S3. Transplanted GCs do not convert into SGCs

Day post transplantation		1	7	28	56
**Percentage of GCs in the allogenic population**	Crayfish 1	96.5%	96.2%	100.0%	100.0%
	Crayfish 2	100.0%	90.0%	100.0%	100.0%
	Crayfish 3	100.0%	96.2%	100.0%	100.0%
